# Resistance to aztreonam-avibactam due to a mutation of SHV-12 in *Enterobacter*

**DOI:** 10.1186/s12941-023-00605-y

**Published:** 2023-06-26

**Authors:** Shikai Wu, Ke Ma, Yu Feng, Zhiyong Zong

**Affiliations:** 1 Center of Infectious Diseases, West China Hospital, Sichuan University, Guoxuexiang 37, Chengdu, 610041 China; 2 Center for Pathogen Research, West China Hospital, Sichuan University, Chengdu, China; 3 Division of Infectious Diseases, State Key Laboratory of Biotherapy, Chengdu, China; 4 Department of Infection Control, West China Hospital, Sichuan University, Chengdu, China

**Keywords:** Resistance, Avibactam, Aztreonam, SHV

## Abstract

Aztreonam-avibactam is an important option against *Enterobacterales* producing metallo-β-lactamases (MBLs). We obtained an aztreonam-avibactam-resistant mutant of an MBL-producing *Enterobacter mori* strain by induced mutagenesis. Genome sequencing revealed an Arg244Gly (Ambler position) substitution of SHV-12 β-lactamase in the mutant. Cloning and susceptibility testing verified that the SHV-12 Arg244Gly substitution led to significantly reduced susceptibility to aztreonam-avibactam (MIC, from 0.5/4 to 4/4 mg/L) but with the loss of resistance to cephalosporins as tradeoff. Arg244 of SHV involves in the binding of avibactam by forming an arginine-mediated salt bridge and is a critical residue to interact with β-lactams. Molecular modeling analysis demonstrated that the Arg244Gly substitution hindered the binding of avibactam to SHV with higher binding energy (from − 5.24 to -4.32 kcal/mol) and elevated inhibition constant Ki (from 143.96 to 677.37 µM) to indicate lower affinity. This substitution, however, resulted in loss of resistance to cephalosporins as tradeoff by impairing substrate binding. This represents a new aztreonam-avibactam resistance mechanism.

*Enterobacter*, a genus of the family *Enterobacteriaceae*, is a group of important human pathogens [1]. Carbapenems are the major choices to treat severe infections caused by *Enterobacter*, but carbapenem-resistant *Enterobacter* has been increasingly reported [1]. Production of metallo-β-lactamases (MBLs) such as NDM, VIM, and IMP is a major mechanism mediating carbapenem resistance in *Enterobacter* [1]. Avibactam (AVI) is a non-β-lactam β-lactamase inhibitor able to inhibit serine-based β-lactamases but not MBLs [2], while aztreonam (ATM) is stable to the hydrolysis by MBLs [3, 4]. The combination of aztreonam-avibactam (ATM-AVI) has activity against *Enterobacter* isolates producing either serine-based β-lactamases or MBLs or both and has been used to treat carbapenem-resistant *Enterobacter* [5, 6]. However, ATM-AVI resistance has also emerged [7–9] but the mechanism for resistance remains largely unknown in *Enterobacter*. In this study, we report a novel mechanism mediating reduced susceptibility to ATM-AVI but with increased susceptibility to cephalosporins as tradeoff.

## An ATM-AVI-resistant mutant was obtained from a carbapenem-resistant *Enterobacter mori* clinical strain

Carbapenem-resistant *Enterobacter* strain 020047 was recovered from urine of a patient in Sichuan, China, in 2016. Genome sequence of 020047 was obtained using a HiSeq X10 sequencer (Illumina, San Diego, CA). Strain 020047 was identified as *Enterobacter mori* based on the draft genome using FastANI v1.33 [10]. Antimicrobial resistance genes were identified using ResFinder (http://genomicepidemiology.org/). Strain 020047 has five β-lactamase-encoding genes, i.e., *bla*_TEM−1B_ encoding a broad-spectrum β-lactamase, *bla*_CTX−M−3_ and *bla*_SHV−12_ encoding extended-spectrum β-lactamases (ESBLs), and two MBL-encoding genes *bla*_IMP−4_ and *bla*_NDM−1_. MIC of ATM-AVI was 1/4 mg/L (Table 1) as determined using broth microdilution according to the Clinical and Laboratory Standards Institute (CLSI) [11]. We conducted multi-step mutant selection experiments as described previously [12] to examine whether 020047 could develop resistance to ATM-AVI and if yes, to investigate the resistance mechanism. Briefly, 10^8^ cfu of strain 020047 was inoculated in 2 mL LB broth (Sigma; St. Louis, MO) containing 0.5/4 mg/L ATM-AVI (0.5 × MIC) and 0.5 mg/L 5-azacytidine (Mce; Shanghai, China), an anticancer drug to increase the mutation rate of bacteria through inducing the SOS reaction [12]. After overnight culture, a 100-µl aliquot was streaked on a LB agar plate with doubled concentrations of ATM from 1 mg/L and fixed 4 mg/L AVI and 0.5 mg/L azacytidine and a colony was collected. The procedure from overnight culture was repeated in each day until ATM reached 16 mg/L as colonies grew in the presence of 8/4 mg/L ATM-AVI were obtained but no colonies could grow in ATM-AVI at 16 mg/L or higher concentrations. An ATM-AVI-resistant mutant, assigned 020047R here, was obtained from the LB agar plate containing 8/4 mg/L ATM-AVI. MIC of ATM-AVI for 020047R was 16/4 mg/L (Table 1) as determined using CLSI broth microdilution [11].

**Table 1:** MICs (mg/L) of antimicrobial agents against strains in this study

Antimicrobial agents	020047	020047R	DH5α::SHV-12	DH5α::SHV-12R
CEP^a^	**> 1024**	**> 1024**	**1024**	**16**
FUR	**1024**	**1024**	**128**	4
CTX	**> 1024**	**1024**	**32**	0.06
PIP-TAZ	**> 512/4**	**> 512/4**	**256/4**	2/4
CAZ	**> 1024**	**> 1024**	**512**	4
CAZ-AVI	**> 512/4**	**> 512/4**	0.25/4	0.25/4
IMP	**16**	**16**	0.12	0.12
ATM	**2048**	**256**	**512**	**32**
ATM-AVI	1/4	**16/4**	0.5/4	4/4
ATM-CLA	**32/16**	**32/16**	1/0.5	2/1
ATM-TAZ	**> 512/4**	**256/4**	**128/4**	2/4
ATM-SUL	**256/128**	**256/128**	**32/16**	8/4

CEP, cephalothin; FUR, cefuroxime; CTX, cefotaxime; PIP-TAZ, piperacillin-tazobactam; CAZ, ceftazidime; CAZ-AVI, ceftazidime-avibactam; IMP, imipenem; ATM, aztreonam; ATM-AVI, aztreonam-avibactam; ATM-CLA, aztreonam-clavulanic acid; ATM-TAZ, aztreonam-tazobactam; ATM-SUL, aztreonam-sulbactam

Those reached the breakpoints to define resistance are highlighted in bold. The breakpoint of ATM-AVI, ATM-CLA, ATM-TAZ, and ATM-SUL to define resistance were according to that of ATM.

^a^Using breakpoints of cefazolin for infections other than uncomplicated urinary tract infection [11]

## A nonsynonymous mutation was identified in *bla*_SHV−12_ in the ATM-AVI- resistant mutant

Like strain 020047, 020047R was also subjected to whole-genome sequencing using HiSeq X10. Reads were assembled using SPAdes v3.14.0 [13] and the genome sequence was annotated using Prokka v1.13 [14]. Single nucleotide polymorphisms (SNP) between 020047 and 020047R were called using Snippy v4.6.0 (https://github.com/tseemann/snippy) and were filtered to remove recombination using Gubbins v2.4.1 [15]. Compared to 020047, 020047R has five SNPs with three in non-coding regions. One SNP was present in a gene encoding a transposase of the Tn*3* transposon family resulting in a Thr to Ala amino acid substitution. The remaining SNP occurred in *bla*_SHV−12_ (C715G, numbered from the ATG start codon) resulting in an Arg to Gly amino acid substitution at position 239 (Ambler position 244, Arg244Gly). By Blast, it becomes evident that among all reported naturally-occurring SHV β-lactamases the Arg244Gly substitution has not been found before. We then focused on the mutation of *bla*_SHV−12_ and performed cloning experiments.

## The Arg244Gly mutation of *bla*_SHV−12_ mediates reduced susceptibility to ATM-AVI but with loss of resistance to cephalosporins as tradeoff

The − 10, and − 35 boxes within the promotor of *bla*_SHV−12_ were predicted using BPROM (http://www.softberry.com/berry.phtml?topic=bprom&group=programs&subgroup=gfindb). Together with the promoter sequence, *bla*_SHV−12_ in 020047 and its variant (assigned *bla*_SHV−12R_ here) in 020047R were amplified with primers SHV-12-PROF (5’-AACCATATGATGATAAGTTTATCACCACCG, with restriction site is underlined) and SHV-12-PROR (5’-AACGAATTCAATACAATCAGGTGGCCAC) using PrimeSTAR Max DNA Polymerase (Takara; Dalian, China). Purified amplicons and the vector pET-28a(+) (Miaolingbio; Wuhan, China) were restricted by *Nde*I and *EcoR*I (Takara, Dalian, China), respectively, and were ligated using T4 ligase (Takara) to construct pET-SHV12 and pET-SHV12R. pET-SHV12 and pET-SHV12R were separately transformed into *Escherichia coli* DH5α using the chemical method [16]. Potential transformants were selected on LB agar plates containing 50 mg/L kanamycin. The presence of *bla*_SHV−12_ or *bla*_SHV−12R_ in the corresponding transformant DH5::SHV-12 and DH5::SHV-12R was verified by PCR using Primers T7 (5’-TAATACGACTCACTATAGGG) and T7ter (5’-TGCTAGTTATTGCTCAGCGG) and subsequent Sanger sequencing.

MICs of cephalothin (CEP), cefuroxime (FUR), cefotaxime (CTX), piperacillin-tazobactam (PIP-TAZ), ceftazidime (CAZ), ceftazidime-avibactam (CAZ-AVI), ATM, ATM-AVI, ATM-clavulanic acid (ATM-CLA), ATM-sulbactam (ATM-SUL), ATM-tazobactam (ATM-TAZ), and imipenem (IMP) against strain 020047, 020047R, DH5::SHV-12 and DH5::SHV-12R were determined using the CLSI broth microdilution [11]. The breakpoints of ATM defined by CLSI were applied for ATM-AVI, ATM-CLA, ATM-SUL, and ATM-TAZ. MIC of ATM-AVI against DH5::SHV-12R was 4/4 mg/L, which was 8-fold higher than DH5::SHV-12 (0.5/4 mg/L, Table 1). However, compared to those against DH5::SHV-12, MICs of cephalosporins were 32- (FUR) to 512-fold (CEP) lower and that of ATM was 16-fold lower against DH5::SHV-12R (Table 1). The above findings suggest that the Arg244Gly substitution of SHV-12 mediates reduced susceptibility to ATM-AVI but leads to loss of resistance to cephalosporins as tradeoff.

## The Arg244Gly substitution of SHV-12 altered the AVI binding pocket and impaired affinity for cephalosporins and ATM

**Fig. 1:**
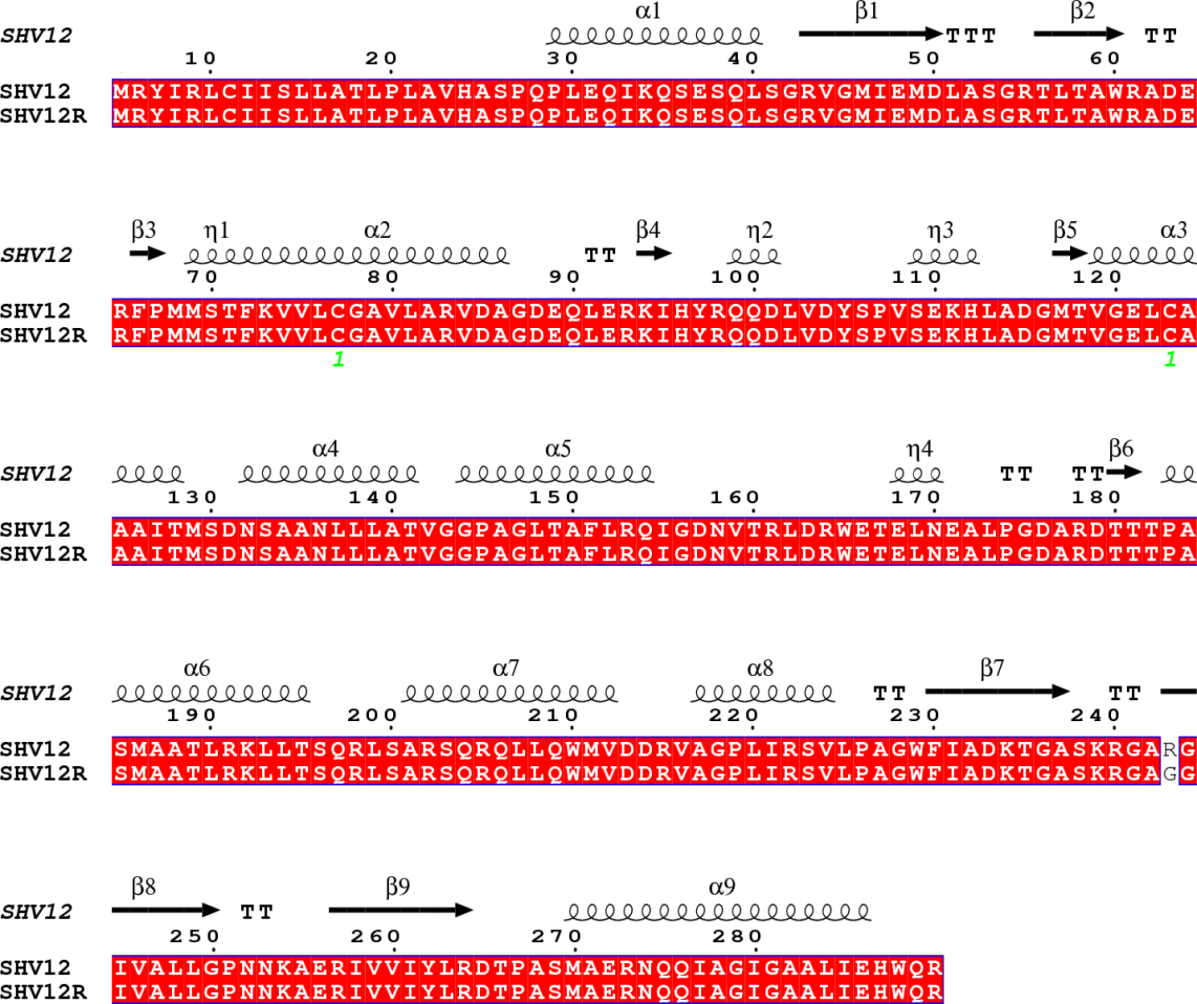
Secondary structure of SHV-12 and SHV-12R. The alignment of amino acid sequences and the prediction of secondary structures were performed using ENDscript 2 [30]. Secondary structure elements, α helixes, β sheets, and 3_10_-helixes (representing by η), are indicated. β-strands are rendered as arrows, and strict β-turns are shown as TT letters

The Arg244Gly substitution occurs in the region to form a β sheet but does not alter the predicted secondary structure of SHV-12 (Fig. 1). The enzyme conformation may change during the process of docking to ligand and there is no crystal structure of SHV-12 β-lactamase bound to AVI in the Protein Data Bank (PDB). The structure of SHV-12 and SHV-12R were predicted using the modelling tool SWISS-MODEL (https://swissmodel.expasy.org/interactive) based on the crystal structure of SHV-1 β-lactamase bound to AVI (PDB: 4ZAM) as template. The ligand, water molecules of SHV-12 and SHV-12R were removed using Pymol (Schrödinger, www.pymol.org). SHV-12 and SHV-12R were then prepared by adding hydrogen atoms with gasteiger charges by AutoDockTools of MGLTools 1.5.6 [17]. The structure of AVI (PubChem CID: 9,835,049) was obtained from the PubChem (https://pubchem.ncbi.nlm.nih.gov/). Ligands were regarded as flexible during docking in default settings using AutoDockTools. AutoGrid and genetic algorithm [18] were used to evaluate the binding energies and intermolecular forces. Grid box was set as 155 × 126 × 126 with a 0.375 Å grid point spacing and default docking parameters. Molecular docking of SHV-12 and AVI was modeled using AutoDock 4.2.6 [17]. The conformation of docking that contained part of known binding sites such as Ser70 [19] (see below) and had lower binding energy and lower inhibition constant Ki (lower Ki means higher affinity), which were determined using AutoDock 4.2.6, was selected. Docking structure was visualized using Pymol (www.pymol.org).

**Fig. 2:**
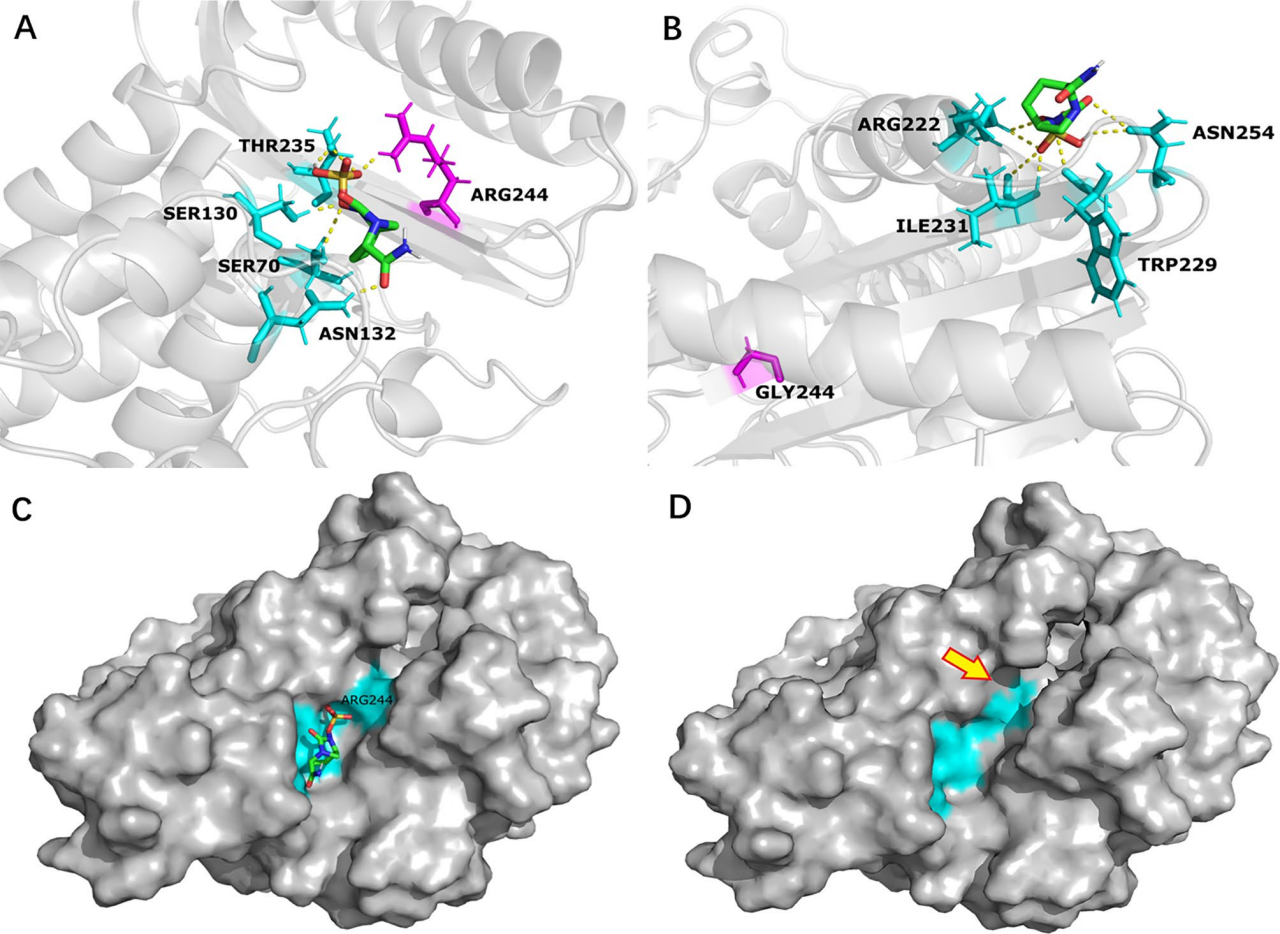
Binding of AVI to SHV-12 and its variant SHV-12R. The residues interact with AVI are depicted in blue. The amino acid substitution is s depicted in purple and the rest of the protein is in green. Molecular docking of SHV-12 and AVI was modeled using AutoDock 4.2.6 [17]. Docking structure was visualized using Pymol (www.pymol.org). **Panel A and C**, hydrogen bonds of AVI to SHV-12. Ser70 is the active site to hydrolyze β-lactams. Arg244 and several other amino acids, e.g., Ser130, Asn132, and Thr235, formed hydrogen bonds (shown as a cyan region in panel C) to AVI. **Panel B and D**, hydrogen bonds of AVI to SHV-12R. The Arg244Gly substitution, indicated by an arrow in panel D, altered hydrogen bonds (shown as a cyan region in panel D) of AVI compared to that in SHV-12 (panel A)

Ser70 (Ambler position, hereinafter) is the active site to hydrolyze β-lactams of SHV-1, SHV-12 and SHV-12R by querying the UniProt database (https://www.uniprot.org/). In previous structure studies [20, 21], Ser70 is covalently bonded to AVI and Arg244 in SHV-1 formed an arginine-mediated salt bridge interacting with the sulfate moiety of AVI. In addition, Arg244 and several other amino acids (Ser130, Asn132, Glu166, Thr167, Asn170, Thr235, and Ala237) of SHV-1 formed hydrogen bonds to AVI (Fig. 2 panel A). In strain 020047R, Arg244 substituted by a shorter Gly239 results in transformation of AVI binding (Fig. 2 panel B), hindering the binding of AVI to SHV-12R. Analysis of binding energy showed that the estimated binding energy increased from − 5.24 kcal/mol in SHV-12 to -4.32 kcal/mol in SHV-12R. The inhibition constant Ki also increased from 143.96 µM in SHV-12 to 677.37 µM in SHV-12R, suggesting lower affinity to AVI. In addition, the pocket accommodating AVI (Fig. 2 panel C) vanished from SHV-12R in the presence of the Arg244Gly substitution (Fig. 2 panel D).

Arg244 of class A β-lactamases including SHV enzymes has been well characterized and is a critical residue to interact with β-lactams and β-lactamase inhibitors, and is involved in recognition, positioning, and turnover of substrates in the active site [22–25]. It has been shown that various amino acid substitutions of Arg244 including Arg244Gly cause that SHV-1 loses the activity of cephalosporinases due to the impaired affinity and are unable to hydrolyze cephalosporins [23, 25]. The hydrolysis of ATM is also reduced but to a lesser extent in the presence of such substitutions of Arg244 [25]. Instead of a carboxylate group in cephalosporins, ATM has a sulfonic acid group bonded to the lactam ring and Arg244 has a weaker interaction with the sulfonic group than that with carboxylate [25]. Although the hydrolysis of ATM by SHV-12R was reduced, it did not completely compensate the effect of the decreased binding of AVI to inhibit the enzyme. As such, the susceptibility of ATM-AVI was significantly reduced for strains producing SHV-12R. By contrast, the reduced hydrolysis of cephalosporins could compensate impaired binding of AVI to make MICs of CAZ-AVI unaltered. However, it is worth to point out that CAZ-AVI alone has no activity against strain 020040 due to the production of MBLs.

Notably, amino acid substitutions of Arg244 have been well described in TEM β-lactamases including TEM-79 (an Arg244Gly variant of TEM-1) [26] and such substitutions lead to resistance to the inhibition of CLA [27, 28]. In the presence of 4 mg/L ATM, However, the activity of ATM-CLA against SHV-12R and SHV-12 was not significantly different (Table 1). Previous studies [23, 29] have found that different amino acid substitutions of Arg244 of SHV-1 obtained by mutagenesis exhibit varied and even contradictory impact on the inhibition of CLA. Particularly, the Arg244Gly substitution of SHV-1 did not significantly (less than four-fold) alter such inhibition [23, 29]. SHV-12R led to a four-fold lower MIC of ATM-SUL (32/4 mg/L) comparing with SHV-12 (Table 1) and this is consistent with the previous finding of 8-fold increased inhibition of SUL against SHV-1 with the Arg244Gly substitution [29]. The impact of Arg244Gly substitution of SHV β-lactamases on TAZ has not been evaluated before, but in this study SHV-12R led to a 64- or 128-fold lower MIC of ATM-TAZ and PIP-TAZ comparing with SHV-12 (Table 1). The above findings highlight that the impact of Arg244Gly substitution varies according to the β-lactamases (e.g., TEM or SHV) and the β-lactamase inhibitors (AVI, CLA, SUL, and TAZ).

We are aware of limitations of this study. First, multiple colonies grew on the agar plate containing 8/4 mg/L ATM-AVI and we only picked up a single colony as the representative for study. We also did not repeat the mutation experiments. We were therefore unable to uncover the presence of other potential mechanisms for mediating ATM-AVI resistance and to determine the reproducibility of the Arg244Gly substitution of SHV-12 to form SHV-12R. Second, we did not determine enzyme kinetics parameters for SHV-12R in comparison with SHV-12, which could provide complementary data to phenotypic, genetic and structural analyses.

Despite the limitations, we identified an amino acid substitution at Arg244 of SHV-12 leading to reduced susceptibility to ATM-AVI, a combination against MBL-producing *Enterobacterales* but with the expense of losing the ESBL phenotype against cephalosporins. This is the first time to the best of our knowledge that a mutation of *bla*_SHV_ is associated with reduced susceptibility to ATM-AVI.

## Data Availability

The draft genome sequences of strain 020047 and 020047R have been deposited into GenBank under the accession no. JAJHUL000000000 and JAJHUM000000000, respectively.
